# *α*-Tocopherol Ameliorates Redox Equilibrium and Reduces Inflammatory Response Caused by Chronic Variable Stress

**DOI:** 10.1155/2018/7210783

**Published:** 2018-11-08

**Authors:** Mariola Herbet, Magdalena Izdebska, Iwona Piątkowska-Chmiel, Monika Gawrońska-Grzywacz, Dorota Natorska-Chomicka, Kamil Pawłowski, Marcin Sysa, Brygida Ślaska, Jarosław Dudka

**Affiliations:** ^1^Department of Toxicology, Medical University of Lublin, Lublin 20-093, Poland; ^2^Department of Biological Bases of Animal Production, University of Life Sciences in Lublin, Lublin 20-950, Poland

## Abstract

Chronic exposure to stress factors contributes to the development of depression by generating excess of reactive oxygen species which leads to oxidative stress and inflammatory processes. The aim of the study was to assess the potential protective properties of *α*-tocopherol supplementation on the rats exposed to chronic variable stress (CVS). Male Wistar rats (50-55 days old, weighing 200-250 g) were divided into three groups (n=10): control, stressed, and stressed and receiving (+)-*α*-tocopherol solution in a dose of 100 mg/kg/day. Rats in the stressed groups were exposed to CVS for 40 days. Markers of redox disorders (glutathione reduced and oxidized levels, GSH/GSSG ratio, glutathione peroxidase, glutathione reductase activities, total antioxidant status, and lipid peroxidation) and inflammatory response (IL-1*β*, IL6, and TNF-*α*) were determined in the blood. Additionally, molecular biomarkers of depression (expression of* Fkbp5 *and* Tph2*) were studied in hippocampus. The biochemical analysis was inconclusive about the presence of oxidative stress in the blood of rats exposed to CVS. However, changes in all parameters suggest presence of redox equilibrium disorders. Similarly, activation of inflammatory processes was observed as a result of CVS. Molecular effects of environmental stress in hippocampus were also observed. Generally, *α*-tocopherol ameliorated redox equilibrium disorders, tempered inflammatory response, and protected from changes in determined molecular markers of depression.

## 1. Introduction

Severe stress leads to dysregulation of the hypothalamic-pituitary-adrenal (HPA) axis and reduced concentrations of serotonin and other neurotransmitters in the brain [1]. Chronic exposure to stress factors therefore contributes, on a global scale, to the development of depression [2]. Major depression is the most common affective disorder that was identified by the World Health Organization as one of leading causes of disability and limitation of work capacity in the world [3]. In the pathomechanism of depression caused by chronic stress, factors such as oxidative stress and inflammatory processes developing in the brain are involved [4–6]. Oxidative stress is defined as an imbalance between the production of reactive oxygen species (ROS) and antioxidant defenses [7]. Studies showed that in the situation of severe stress factors activity, increase of the energy demands (accelerated glucose metabolism) is present, which results in intensified generation of reactive oxygen species (ROS) [8]. Reactive oxygen species, such as superoxide anions, nitric oxide, and hydrogen peroxide, are highly reactive and are naturally formed as byproducts of electron transportation and energy metabolism. In order to defend against ROS, cells developed several antioxidant defense systems. A variety of antioxidant enzymes, such as superoxide dismutase, catalase, and glutathione-S-transferase, can facilitate reduction reactions of ROS. However, increased ROS generations combined with insufficient antioxidant activity can lead to oxidative damage. Oxidative stress induces lipid peroxidation, damage to biological membranes, proteins, DNA, and RNA oxidation, and consequently the brain structural degeneration, metabolism disorders, and impaired functioning of the hippocampus and the prefrontal cortex observed in the development of depression [9].

Chronic stress proved to be associated with elevated circulating levels of inflammatory biomarkers [10, 11]. Neuroinflammation is closely related to excessive oxidative stress and is described as a major factor responsible for the development and progression of depressive disorder associated with chronic stress. Redox status plays an essential role in the modulation of inflammation and immune response. During inflammation, the production of intracellular reactive oxygen species is involved in triggering inflammatory responses via the secretion of proinflammatory mediators, notably prostaglandins and leukotrienes, and cytokines like tumor necrosis factor alpha (TNF-*α*) and interleukin-6 (IL-6), which in turn increases inflammation and can lead to excessive damage to brain tissue [12].

Depressive disorder is very difficult to diagnose until symptoms intensify. Unfortunately, there are still no laboratory or imaging tests that would help diagnose this disease. Diagnostic criteria for major depressive episode are based on the psychological examination and the elimination of other disorders as diagnoses. Therefore, it seems advisable to conduct research on the development of biomarkers of depression, because it is a disease that requires pharmacological treatment. The current pharmacotherapy of depression, although it is becoming more and more effective, does not provide satisfactory results. The drugs used often give a therapeutic effect only after few weeks. Moreover, they are associated with the occurrence of undesirable effects and can lead to the development of drug-resistant depression, which makes further pharmacotherapy questionable. Alpha-tocopherol is a potent antioxidant especially with lipoperoxyl radical scavenging activities and may help reduce free radical damage of brain. In addition to its activities as an antioxidant, *α*-tocopherol has anti-inflammatory properties and is commonly used to regulate inflammatory diseases among others by inhibiting cyclooxygenase-2 (COX-2) and 5-lipoxygenase (5-LOX) involved in the formation of eicosanoids (prostaglandins and leukotrienes) [13]. The studies showed that *α*-tocopherol also inhibited LPS-stimulated IL-6 in macrophages [14].

Considering the above, the aim of our study was to assess the potential preventive effect of *α*-tocopherol supplementation on the markers of oxidative stress and inflammation in the blood of rats after exposure to chronic variable stress (CVS). Understanding the mechanisms presumably involved in the protective role of *α*-tocopherol in chronic stress may help to more precisely define the clinical situations where its supplementation will prove to be beneficial. The chronic variable stress is a well-validated animal model of depression, reflecting behavioral and neurochemical disorders that occur in the humans with depression caused by chronic stress [15–17]. To confirm the depressive changes induced by CVS and to understand the molecular mechanisms in the brain of rats, we also evaluated two important genes used as molecular indicators of depression:* Fkbp5* (FK506 binding protein 5) and* Tph2 *(tryptophan hydroxylase 2). FKBP5 has been identified as a candidate gene for major depressive disorder stress-related conditions because it interacts with the HPA axis and plays a role in the regulation of neurobiological stress responses [2, 18, 19]. The studies found that in individuals with the presence of alleles of this gene responsible for increased expression of FK506 binding protein 5, a more prolonged cortisol response to psychological stressors was observed. This altered responsiveness to stress seems to predispose to psychiatric disorders; a number of studies linked FKBP5 polymorphisms with an increased susceptibility to major depression [20–22]. The* Tph2* gene may be a reliable biomarker for major depression because the levels of TPH2 mRNA and protein expression were consistently found to be elevated in depressed suicide victims [23]. To assess the redox status, the glutathione concentrations (GSH, GSSG, and GSH/GSSG ratio), antioxidant enzyme activities (glutathione peroxidase GPx and glutathione reductase GR), total antioxidant status (TAS), and thiobarbituric acid reactive substances (TBARS as an index of lipid peroxidation) were measured. We also investigated the effect of CVS on the concentration of major cytokines associated with depression, i.e., IL-1*β*, IL6, and TNF-*α* in the blood of rats.

## 2. Materials and Methods

### 2.1. Animals and Housing

The study was carried out on male Wistar rats (weighing initially 200-250 g, approximately 50-55 days old) from a licensed breeder (Brwinów, Poland). The animals were maintained under standard laboratory conditions (temp. 20 ± 2°C, constant humidity of 55 ± 10%, noise) on a 12 h day/12 h night cycle with free access to food and water. The rats were allowed to habituate to the new living environment for five days. The experiment was approved by the First Local Ethics Committee on Animal Experimentation of the Medical University of Lublin, Poland (permit no. 12/2015). All procedures were conducted in accordance with the European Communities Council Directive of 22 September 2010 (2010/63/EU) and Polish legislation acts concerning animal experimentations with every effort made to minimize the suffering of animals.

### 2.2. Chronic Variable Stress (CVS) Procedure

Chronic variable stress (CVS) procedure was carried out as described by Gamaro et al. with slight modifications [15, 24]. The rats were randomly divided into three groups (I-III), each consisting of 10 animals (the optimal sample size in order to conduct a proper statistical analysis, for sufficient statistical power): I, CTL (control group, not subjected to stressors, standard breeding conditions, 5 rats in each home cage of dimensions 65x25 cm, 18 cm high); II, CVS (stressed); and III, CVS+*α*-tocopherol (CVS+*α*-TF) (stressed and receiving (+)-*α*-tocopherol solution from vegetable oil (type V, ~1000 IU/g; Sigma-Aldrich, Poland) in a dose of 100 mg/kg b.w (1 IU = 0.67 mg of (+)-*α*-tocopherol, prepared in corn oil)* per os* by probe, in volumes of 0.5 ml/100 g b.w. for 40 days).

According to the CVS experimental protocol, the rats were exposed to various, unpredictable (at different times intervals) stress conditions for 40 days (Table 1) [24].

In the experiment, the following stressors were used: 24 h of water deprivation; 24 h of food deprivation; 1–3 h of restraint (each rat was placed inside a 26 × 6 cm plastic tube followed by tape adjustments on the outside to prevent it from moving, and breathing was enabled through a 1 cm hole at the far end of the tube); 1.5–2 h of restraint at 4°C (in the same tube); forced swimming for 10 or 15 min (was performed by placing the rat in a round glass tank of 50 cm radius filled with 23°C water); flashing light for 120–210 min (each rat was put inside a 50 cm high open-field container 40 × 60 cm, made of brown plywood with a frontal glass wall; a flashing light was delivered from a 40W lamp, set at 60 flashes/min); and isolation for 24 h.

After 40 days of stress procedures and 24 h after the last administration of *α*-tocopherol, the rats were decapitated. The brain from each animal was obtained and washed with 20 ml of saline. The samples of isolated rats hippocampus were rinsed with 20 *μ*l of saline and stored at –75°C before the process of isolation of RNA. The blood samples were taken and divided as follows: one portion was stored in a heparin tube (whole blood) and the other was left to clot. The whole heparinized blood (for quantitative in vitro determination of GPx in whole blood) was tested to estimate the GPx activity. In the portion of the blood that was allowed to clot, the serum fraction was removed and the glutathione reductase GR activity, the levels of glutathione (GSH and GSSG), total antioxidant status (TAS), thiobarbituric acid reactive substances (TBARS), IL-1*β*, IL6, and TNF-*α* in the serum fraction were determined.

### 2.3. The Quantitative Real-Time PCR Analysis (qPCR)

According to the manufacturer's instructions, the nucleic acid was separated from tissue using Syngen Tissue RNA Mini Kit (Syngen Biotech, Poland), and reverse transcription using NG dART RT-PCR kit (EURx, Poland) was performed. The assessment of the selected genes expression (*Fkbp5 *and* Tph2*) was performed using a quantitative real-time PCR (qPCR) method. Gene symbols, gene names, GenBank reference sequence accession numbers, and assay IDs used in this study for qPCR are presented in Table 2.

To determine the relative levels of* Fkbp5 *and* Tph2* expression, the ΔΔCt method was used. The results are shown as RQ value. The level of expression was calculated with reference to a* Hprt* gene that has a constant expression level in the tissue and is a housekeeping gene responsible for nucleotide metabolism. The difference between the Ct value of the tested genes and the Ct value of the reference gene was calculated. The reaction was carried out in octuplicate by q-PCR using the SmartChip Real-Time PCR System (WaferGen Biosystems) and TaqMan Fast Universal PCR Master Mix (2x) (Applied Biosystems, USA) according to manufacturer's instructions. Sample quality screening based on amplification, Tm, and Ct values was performed to remove any outlier data points before ΔΔCt calculation and to determine fold change in mRNA levels. The data was presented as RQ value (RQ = 2^-ΔΔCt^).

### 2.4. Determination of Biochemical Parameters

The determinations of oxidative stress parameters: concentrations of GSH, GSSG, and GSH/GSSG ratio, antioxidant enzyme activities (glutathione peroxidase (GPx) and glutathione reductase (GR)), total antioxidant status (TAS), lipid peroxidation (thiobarbituric acid reactive substances), and the concentrations of cytokines IL-1*β*, IL6, and TNF-*α* were measured in the blood of rats by ready-to-use diagnostic kits: GSH/GSSG Ratio Assay Kit (Calbiochem®, a registered trademark of Merck, Darmstadt, Germany), Glutathione Peroxidase Assay Kit and Glutathione Reductase Assay Kit (Cayman Chemical, Ann Arbor, USA), TAS (RANDOX Laboratories Ltd. Crumlin, United Kingdom), TBARS Assay Kit (Cayman Chemical, Ann Arbor, USA), IL-1*β* (ELISA Kit for Interleukin 1 Beta, Cloud-Clone Corp., USA), IL-6 (Murine IL-6 ELISA Kit Diaclone SAS, France), and TNF-*α* (Murine TNF-*α* ELISA kit, Diaclone SAS, France). All diagnostic procedures were performed according to the instructions supplied by the manufacturers of the respective kits.

### 2.5. Statistical Analysis

The results were analyzed statistically in STATISTICA 12.0 application (StatSoft, Cracow, Poland). Data were expressed as mean ± SEM (standard error of the mean). Statistical significance among groups was determined using two-way ANOVA with a* post hoc* Bonferroni multiple comparison test.* P* values less than 0.05 were considered significant.

## 3. Results

### 3.1. The Level of mRNA Expression for* Fkbp5 *and* Tph2* Genes (qPCR Analysis)

We evaluated the relative expression of two important genes used as molecular indicators of depression as a consequence of exposure to CVS:* Fkbp5 *(FK506 binding protein 5) and* Tph2* (tryptophan hydroxylase 2). Sample variation was accounted for by comparison to the expression levels of* Hprt*, which is a housekeeping gene responsible for nucleotide metabolism.

**Figure 1:**
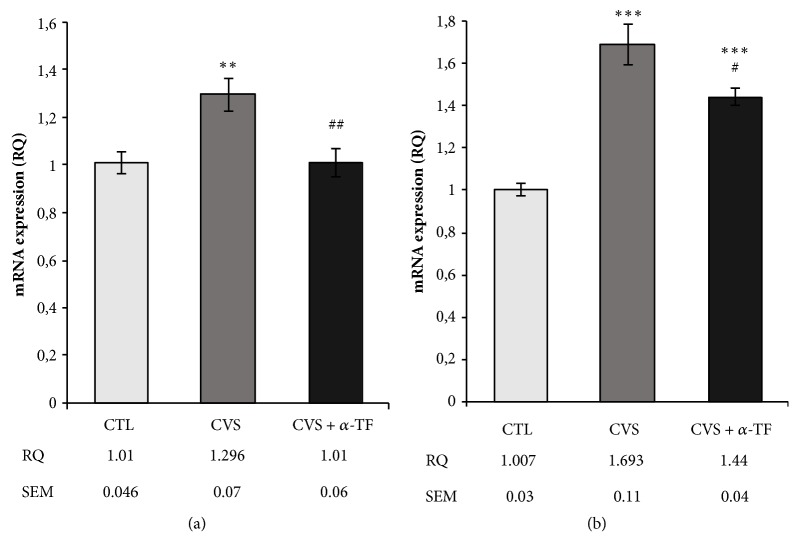
The impact of chronic variable stress and the effects of *α*-tocopherol action on the level of mRNA expression for* Fkbp5* (a) and* Tph2* (b) genes in hippocampus of rats. The mRNA's expression was assayed in regard to the control group and estimated as RQ value (RQ = 2^-ΔΔCt^). Data is displayed as mean ± SEM. Significance: *∗∗ p *< 0.01, *∗∗∗ p* < 0.001 compared to CTL; #* p *< 0.05, ##* p* < 0.01 compared to CVS group (two-way ANOVA with a* post hoc* Bonferroni multiple comparison test).

Expression of mRNA was measured with reference to the control group and was estimated as RQ value (RQ = 2^-ΔΔCt^). In the hippocampus of rats submitted to CVS, the mRNA levels of* Fkbp5 *(F_[3.23]_ = 7.294,* p *< 0.01; Figure 1(a)) and* Tph2* (F_[3.23]_ = 25.969,* p* < 0.001; Figure 1(b)) were significantly increased in comparison to the control. Alpha-tocopherol significantly decreased the level of mRNA expression for* Fkbp5* (F_[3.23]_ = 7.294,* p* < 0.01; Figure 1(a)) and* Tph2* (F_[3.23]_ = 25.969,* p* < 0.05; Figure 1(b)) genes in hippocampus of rats exposed to CVS procedure compared to the CVS group.

### 3.2. GSH and GSSG Levels

**Figure 2:**
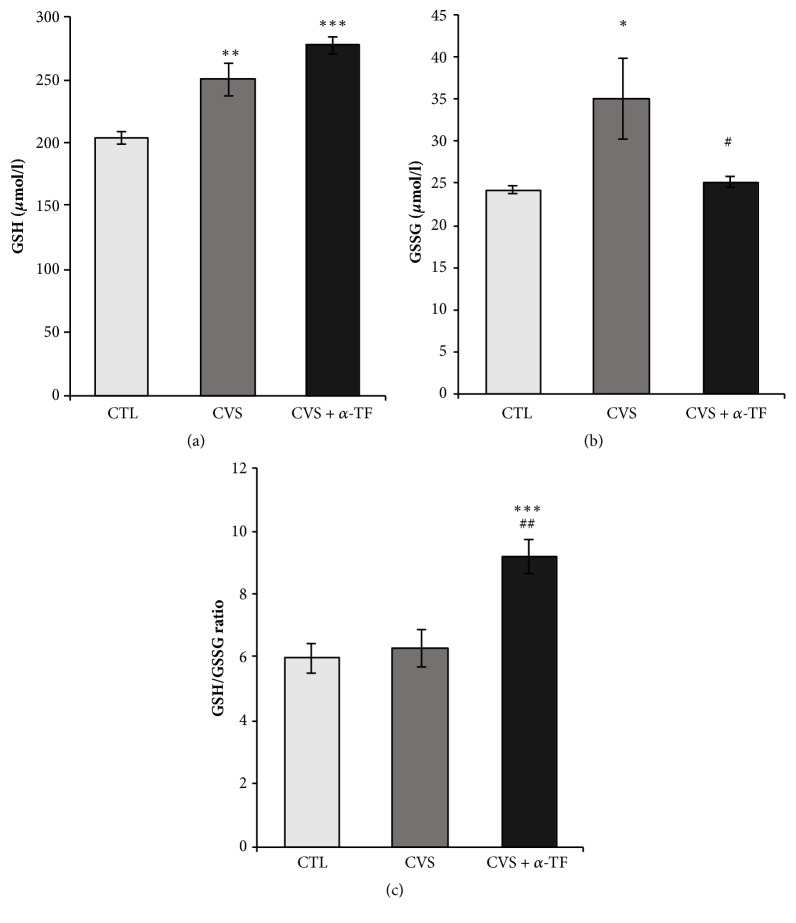
The effects of chronic variable stress (CVS) and *α*-tocopherol action on the GSH (a) abd GSSG (b) concentrations and the GSH/GSSG ratio (c) in the blood of rats. The results are presented as mean ± SEM (standard error of the mean). Significance: *∗ p *< 0.05; *∗∗ p* < 0.01; *∗∗∗ p* <0.001 compared to CTL group; #* p *< 0.05; ## p < 0.01 compared to CVS group (two-way ANOVA with a* post hoc* Bonferroni multiple comparison test).

A significant increase in the concentrations of glutathione GSH (F_[3.35]_ = 17.544,* p* < 0.01; Figure 2(a)) and GSSG (F_[3.35]_ = 4.601,* p *< 0.05; Figure 2(b)) in the blood of rats exposed to CVS compared to the control group was found. However, chronic variable stress did not increase the GSH/GSSG ratio (sensitive indicator of the redox state) in the blood of rats, which suggests that oxidative stress did not yet develop (Figure 2(c)). Alpha-tocopherol (100 mg/kg po) administrated to rats exposed to CVS (CVS + *α*-tocopherol group) decreased the GSSG concentration (F_[3.35]_ = 4.601,* p *< 0.05; Figure 2(b)) and increased the GSH/GSSG ratio (F_[3.35]_ = 11.436,* p* < 0.01; Figure 2(c)) compared with the group of animals only subjected to CVS. Alpha-tocopherol also increased the GSH concentration (F_[3.35]_ = 17.544, p < 0.001; Figure 2(a)) and GSH/GSSG ratio (F_[3.35]_ = 11.436,* p* < 0.001; Figure 2(c)) in comparison to the control group (CTL).

### 3.3. Glutathione Peroxidase (GPx) and Glutathione Reductase (GR) Activity

**Figure 3:**
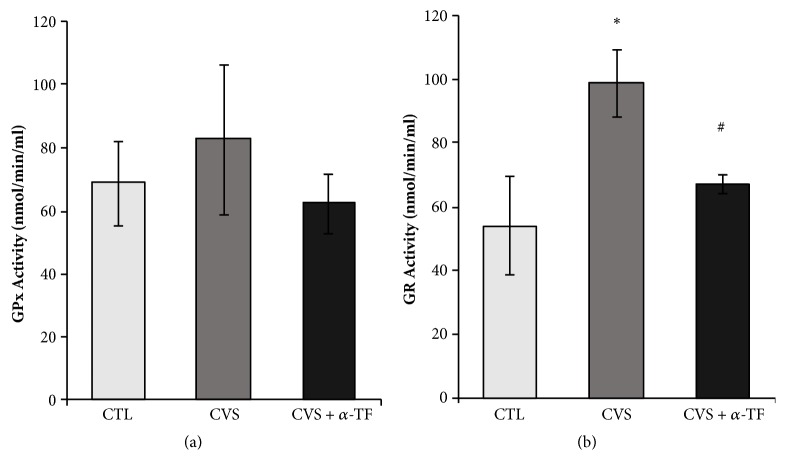
The effects of chronic variable stress and *α*-tocopherol action on the glutathione peroxidase (a) and glutathione reductase (b) activity in the blood of rats. The results are presented as mean ± SEM (standard error of the mean). Significance: *∗ p* <0.05, compared to CTL group; #* p* < 0.05, compared to CVS group (two-way ANOVA with a* post hoc* Bonferroni multiple comparison test).

No statistically significant changes in the glutathione peroxidase (GPx) activity were observed in experimental groups compared to control (Figure 3(a)). However, the results indicated that the CVS significantly increased the activity of glutathione reductase (GR) in the blood of rats in comparison with the CTL group (F_[3.35]_ = 4.521,* p* < 0.05; Figure 3(b)). Alpha-tocopherol administrated to rats exposed to CVS decreased the GR activity (F_[3.35]_ = 4.521,* p* < 0.05; Figure 3(b)) compared to CVS group.

### 3.4. Total Antioxidant Status (TAS) Concentration

**Figure 4:**
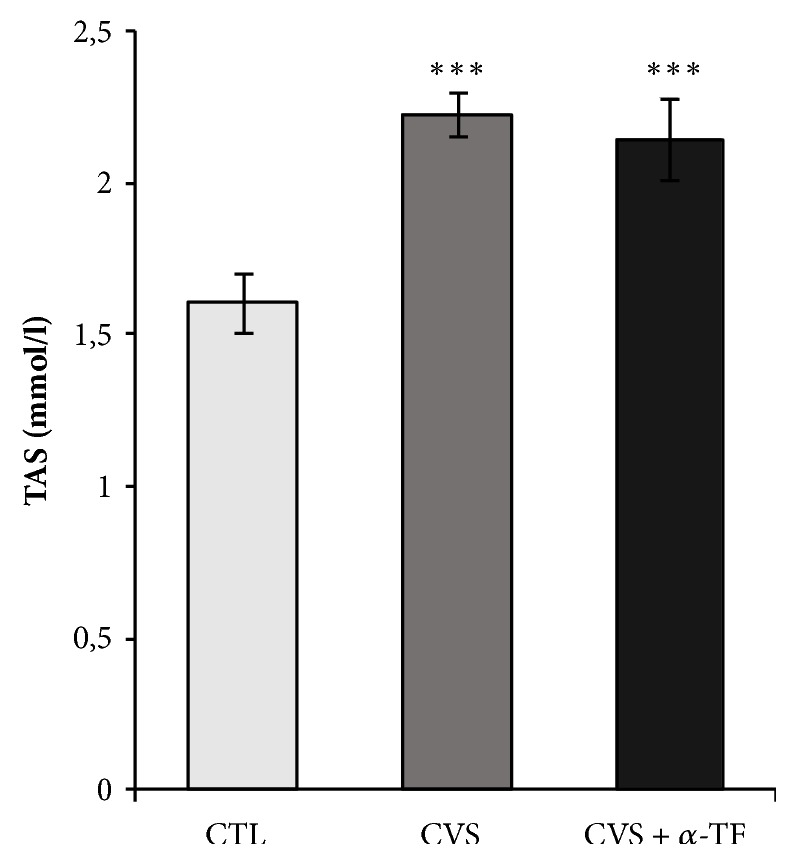
The effects of chronic variable stress and *α*-tocopherol action on the total antioxidant status (TAS) concentration in the blood of rats. The results are presented as mean ± SEM (standard error of the mean). Significance: *∗∗∗ p *< 0.001, compared to CTL group (two-way ANOVA with a* post hoc* Bonferroni multiple comparison test).

We noticed that the chronic variable stress increased the TAS concentration in the blood of CVS rats compared to the CTL group (F_[3.35]_ = 22.579,* p* < 0.001; Figure 4). Alpha-tocopherol did not affect the TAS concentration in the blood of rats (CVS + *α*-tocopherol group) in comparison with CVS group.

### 3.5. Thiobarbituric Acid Reactive Substances (TBARS) Concentration

**Figure 5:**
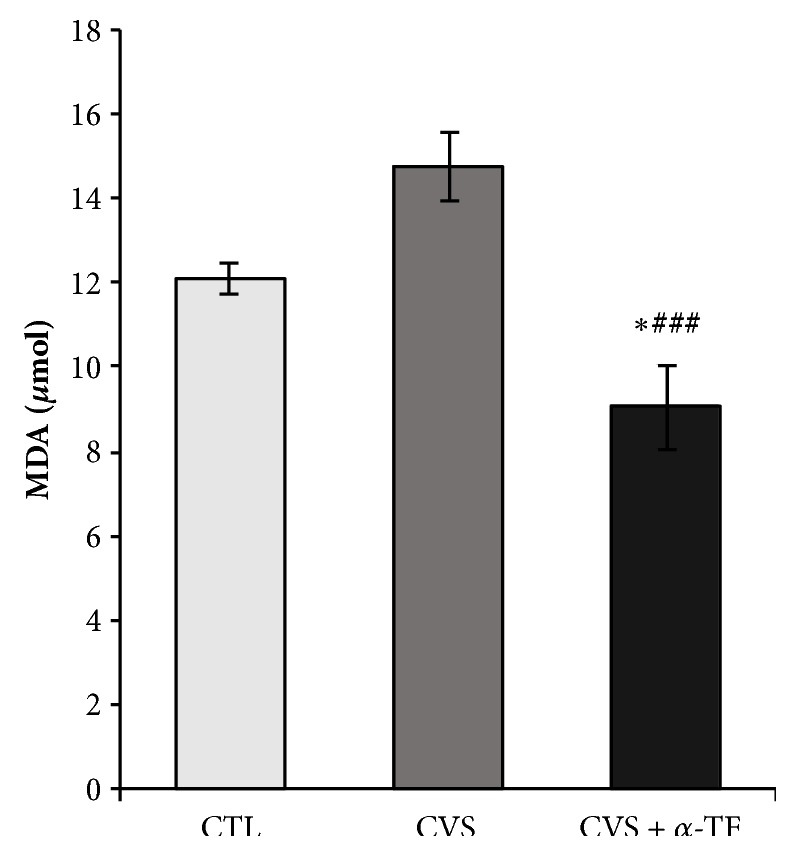
The effects of chronic variable stress and *α*-tocopherol action on the thiobarbituric acid reactive substances (TBARS) by measuring malondialdehyde (MDA) concentration in the blood of rats. The results are presented as mean ± SEM (standard error of the mean). Significance: *∗ p *< 0.05, compared to CTL group; ###* p* < 0.001 compared to CVS group (two-way ANOVA with a* post hoc* Bonferroni multiple comparison test).

In this experiment, we evaluated thiobarbituric acid reactive substances, as a marker of lipid peroxidation, by measuring malondialdehyde (MDA) concentration present in the samples. It was found that *α*-tocopherol significantly decreased the level of malondialdehyde in the blood of rat exposed to CVS procedure (F_[3.35]_ = 13.786,* p* < 0.001; Figure 5) compared to the CVS group. In the group of rat exposed to CVS and receiving *α*-tocopherol there was also a significant reduction in MDA concentration compared to control (F_[3.35]_ = 13.786,* p* < 0.05; Figure 5).

### 3.6. The Concentrations of Cytokines: IL-1*β*, IL-6, and TNF-*α*

**Figure 6:**
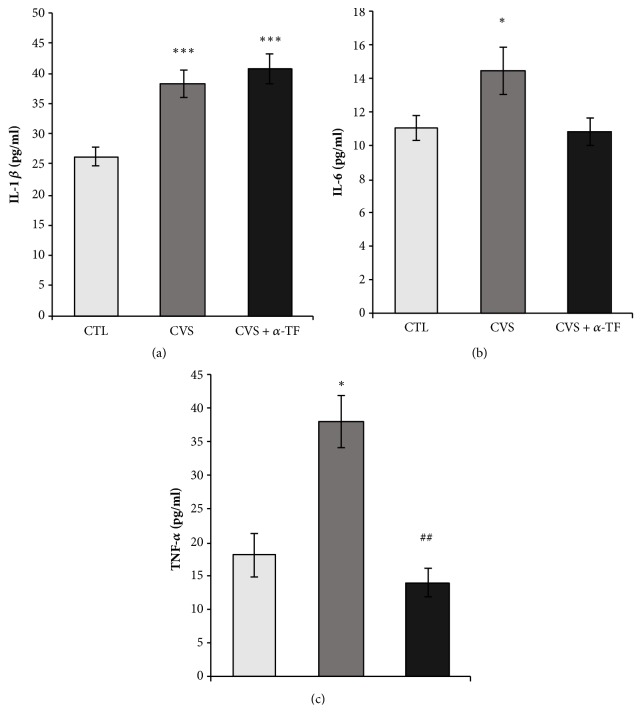
The impact of chronic variable stress and the effects of *α*-tocopherol action on the concentrations of IL-1*β* (a), IL-6 (b), and TNF-*α* (c) in the blood of rats. Data is displayed as mean ± SEM. Significance: *∗ p* < 0.05; *∗∗∗ p* <0.001 compared to CTL group; ##* p* < 0.01 compared to CVS group (two-way ANOVA with a* post hoc* Bonferroni multiple comparison test).

Current study showed that the chronic variable stress (CVS) increased the concentration of IL-1*β* (F_[3.35]_ = 14.766,* p* < 0.001; Figure 6(a)), IL-6 (F_[3,715]_ = 3,854* p* < 0.05 Figure 6(b)), and TNF*α* (F_[3.35]_ = 6.986,* p* < 0.05; Figure 6(c)) compared to CTL group. Alpha-tocopherol significantly decreased only the concentration of TNF*α* in the blood of rat exposed to CVS procedure (F_[3.35]_ = 6.986,* p* < 0.01; Figure 6(c)) compared to the CVS group.

## 4. Discussion

The exposure to chronic stress can be considered as a major environmental factor responsible for the development of depression. The stress activates the hypothalamus-pituitary-adrenal (HPA) axis and leads to glucocorticoid (GC) release from the adrenal glands. The activation of the HPA axis is controlled through a negative feedback mechanism, by the activation of glucocorticoid receptors (GRs). Under stress conditions, the function of the HPA axis is disrupted which can lead to structural and functional changes in the brain [2]. Studies revealed that dysregulation of steroid hormone receptors can cause depressive disorders; the impaired GRs sensitivity in major depression was often reported [20]. FKBP5 regulates GRs sensitivity, lowering the affinity of receptor for its ligand and reducing the efficiency of the nuclear translocation [2]. In this study, the authors provided evidence that animals expressed alteration in the mechanisms controlling GR translocation (which is important for HPA axis function and GRs activity) after exposure to chronic variable stress. Increased expression of* Fkbp5* was observed in the hippocampus of rats exposed to chronic stress. This is in line with previous studies which confirm that FKBP5 gene is activated by stress [25]. A probable consequence of these changes may be an impaired HPA axis feedback inhibition as well as GRs resistance, which may be suggestive of an impaired ability of the receptor to translocate to the nucleus and activate GRs-dependent transcriptional mechanisms. Other evidence confirming the presence of depressive state in rats after exposure to CVS is also the significant increase in the expression of* Tph2* mRNA in our experiment. Clinical studies confirm that there is a connection between TPH2 genetic polymorphisms and depression and suggest that elevated TPH2 expression and activity may be a biomarker or endophenotype of depression [26]. The levels of* Tph2* mRNA expression were consistently found to be elevated in depressed suicide victims [23]. TPH2 is specifically expressed in the serotonergic neurons of the brainstem raphe complex and it was identified as a neuronal-specific isoform which controls brain serotonin (5-hydroxy-tryptamine, 5-HT) synthesis [27, 28]. 5-HT regulates GRs expression and stress hormone release, and it contributes to early-life programming of the plasticity of HPA axis response to stress [29]. Increase of* Tph2* gene expression after chronic stress conditions probably remains in connection with the stress-induced alteration in glucocorticoid levels.

The oxidative stress is one of the possible mechanisms underlying neurodegeneration [30]. Under physiological circumstances, excess of ROS is eliminated by cellular enzymatic and nonenzymatic antioxidant mechanisms. However, under pathological circumstances, there is a shift towards a prooxidative state due to the increase in prooxidation markers and/or reduction in antioxidant mechanisms [30]. The persistent increase of oxidative stress leads to cellular damage manifested through lipid peroxidation, protein, and DNA damage. There is an increasingly growing interest in identifying biomarkers for diseases, in which oxidative stress is involved. In the present study, blood levels of reduced glutathione (GSH), oxidized glutathione (GSSG), antioxidant enzyme activities (GPx and GR), TAS, and TBARS were determined. As current study revealed, a statistically significant increase in GSH, GSSG, and GR levels in the blood of rats exposed to chronic variable stress in comparison to the group not subjected to the stress factors was recorded. Glutathione is an intracellular peptide with various functions such as detoxification, antioxidant defense, and regulation of cellular proliferation. Glutathione, together with its related enzymes (GPx, GR), constitutes a system that maintains the intracellular reducing environment [31, 32]. The oxygen radical scavenging activity of glutathione directly facilitates ROS neutralization and the repair of ROS-induced damage. GPx catalyzes the reduction of organic hydroperoxides and hydrogen peroxide by using glutathione as the reducing agent. GR plays a critical role by regenerating reduced glutathione from the oxidized form. Increase in GSSG observed in our study indicates an increase in ROS generation after chronic stress conditions. However, it should be noted that simultaneously in this group of animals (CVS) there was an increase in GSH level, and no changes in GSH/GSSG ratio were observed. The GSH/GSSG ratio tends to decrease in severe oxidative stress and the accumulation of GSSG, which in turn can lead to decreased defense against free radicals [33]. The consequence of oxidative stress is the observed increase in GSSG level with a concomitant decrease in GSH. Considering the above and also the significant increase in GR activity in this group, it can be assumed that the above results do not confirm the occurrence of oxidative stress in the blood of rats after exposure to CVS. Interestingly, the same markers of oxidative stress were previously determined in the prefrontal cortex of stressful rats [24]. The results of quoted study showed a decrease of the GSH, GSH/GSSG ratio and increase of the GSSG and provided indirect evidence of ROS overproduction and presence of disrupted oxidative defense systems. An important difference is, in this case, a greater concentration of GSH in whole blood than in the brain. One of the reasons for lowered GSH concentration in the brain may be a decrease in its synthesis. The speed of glutathione synthesis depends mainly on the availability of its precursors—cysteine and methionine. Other studies proved that under chronic stress, the level of amino acids in the brain is reduced, because they can be a source of energy production in gluconeogenesis [34]. To summarize, biochemical analysis does not confirm the occurrence of oxidative stress in the blood of rats exposed to CVS; there was no indication that the antioxidative defense barrier was broken, because there was no increase in lipid peroxidation products. Results obtained for GSH, GSSG, GSH/GSSG, GPx, GR, and TAS suggest adaptive reaction mechanism for redox equilibrium disorders in CVS rats; observed decrease in GSSG requires higher activity of GR that replenishes GSH (adaptive higher level).

The results of our presented research may also indicate the effectiveness of the antioxidant system in the whole blood of stressed rats. In addition to the increased GR activity, we also noted a significantly increased TAS level. GR is an especially important enzyme in erythrocytes, which protects against hemolysis. TAS is a marker of overall antioxidant capacity, which reflects the dynamic equilibrium between different prooxidants and antioxidants in blood [35]. In response to overproduction of ROS, TAS might be elevated as a compensatory response to reestablish redox homeostasis [36]. An increase in TAS value may indicate an increasing amount of endogenous antioxidants in the whole blood. It should be noted that the antioxidant capacity of an organism depends also on the diversity and activity of proteins with antioxidant properties. Ceruloplasmin, ferritin, transferrin, and albumin are examples of proteins which are responsible for protection against free radical formation in the blood [37]. The antioxidant properties in the blood are also exhibited by uric acid, which in the presence of ascorbate captures the hydroxyl radical [38]. In our work we also marked TBARS, biomarker of oxidative damage to lipids. TBARS assay measures lipid peroxidation end product—malondialdehyde (MDA), a reactive aldehyde produced from polyunsaturated fatty acids. The study showed no changes in the level of MDA in the blood of rats after exposure to the CVS compared to control groups. These data are coherent with the above results and does not indicate the presence of oxidative stress in whole blood.

Recent evidence indicates that oxidative stress and inflammation pathways are inseparably interconnected and they can play important roles in the pathophysiology of neurodegenerative diseases [39, 40]. The nervous and immune systems interact to maintain physiological homeostasis during inflammation and stressors that induce systemic cytokine production [5, 11]. Chronic stress may induce an inflammatory response with increased production of proinflammatory cytokines, such as IL-6, IL-1*β*, and TNF-*α* [39]. TNF is a key cytokine that initiates and promotes inflammation. IL-6 is a small multifunctional protein that can be released from a myriad of tissues including white blood cells [41]. IL-1*β* is a critical mediator of adaptive stress response as well as stress-associated neuropathology. Our results show that rats after exposure to chronic stress have increased levels of IL-1*β*, IL-6, and TNF-*α* in the serum. The relationship between the interleukins (ILs) alteration and chronic stress is most likely related to corticotrophin-releasing hormone (CRH) that directly stimulates IL-6, IL-1, and IL-12. The induction of inflammatory mediators, in particular cytokines such as IL-6 and TNF, contributes to the generation of ROS under proinflammatory conditions which can result in enhanced cell damage and thus aggravates neurodegeneration [42]. However, the interaction between ILs and the HPA axis is bidirectional. Released cytokines demonstrate the ability to stimulate the HPA axis, and on the other hand glucocorticoids suppress the production of ILs [43]. Stress-induced ILs-mediated secretion of glucocorticoids alters various neurobehavioral processes; in particular, IL-1 plays an important role in stress-induced modulation of memory functioning. Elevation in IL-1 levels mediates the effects of stressors on hippocampal neurogenesis, which may be involved in depressive behavior [43]. The elevation in circulating cytokines' levels is one of the consistent biomarkers of depression [44]. A significant increase in plasma IL-6, IL-1*β*, and TNF-*α* concentrations, indicating the inflammatory processes in major depressive disorder, thus supporting the cytokine hypothesis of major depression, was recorded in many studies [45, 46]. The results observed in our study are consistent with the data presented in the literature and confirm the relationship between chronic stress and inflammatory response. It should be noted that inflammation is a protective response to stress, so the increase of proinflammatory cytokines causes reorganization of tissues. However, uncontrolled inflammation can cause excessive cell damage, which is especially relevant to chronic exposure to stress.

Finally, we provide evidence that chronic *α*-tocopherol supplementation is able to prevent, at least partially, the alterations caused by the CVS exposure. Oxidative stress activates a variety of inflammatory mediators involved in neurodegenerative diseases. Many studies demonstrated that *α*-tocopherol protects tissue against oxidative damage and shows anti-inflammatory effect; however, human research has yielded limited results [13, 14, 47]. Alpha-tocopherol protects lipids and lipoproteins against cell membrane oxidation and maintains an appropriate oxidation-reduction potential. Its main role is to scavenge free organic radicals, terminate lipid peroxidation reactions, and suppress singlet oxygen. Lowering its content in biological membranes, e.g., in erythrocytes, contributes to the intensification of lipid peroxidation processes and the increase in membrane permeability. The anti-inflammatory effects of *α*-tocopherol result from actions on several enzymes involved in inflammatory pathways, such as protein kinase, protein phosphatase 2A, 5-, 12-, and 15-lipoxygenases, phospholipase A2 (PLA2), and COX-2 [14].

In the present study, the analysis of the expression of the* Fkbp5 *and* Tph2* genes indicates the occurrence of a depressive state in the brain of rats after exposure to chronic variable stress. Interestingly, animals that received *α*-tocopherol when exposed to stressors presented reduced genes expression compared to the stressed group. In addition, it was noted that mRNA level of* Fkbp5* remained at the level of nonstressed animals. A large body of evidence indicates that negative feedback control of CRH secretion may be impaired because of altered GRs function in the hippocampus, the key region for major depression and stress-related disorders [48]. It may be inferred that *α*-tocopherol probably affects GR receptor activation and that response to treatment is associated with a normalization of GR function. In line with our results, several studies provide evidence that in the brain antidepressants enhance GC sensitivity and thereby restore GRs-mediated feedback inhibition on the HPA axis [2]. Studies showed that the state of receptor phosphorylation may have a role in controlling activation, gene transcription, and turnover of GRs [49]. The receptor can be phosphorylated as a consequence of ligand binding but it may also occur through the activation of different signaling pathways, including protein kinase A, cyclin-dependent kinase, which may cooperate to modulate GRs sensitivity and function [2]. It can therefore be assumed that the mechanism underlying the influence of alpha-tocopherol on the regulate GRs function is the modulation of its phosphorylation through the action on enzymes and proteins involved in inflammatory pathways; however, more detailed studies are required to confirm this hypothesis. Data suggests that prolonged stress exposure elevated* Tph2* expression [23]. Tryptophan hydroxylase 2 (TPH2) is the rate-limiting enzyme in the synthesis of neuronal 5-HT (controls the brain serotonin concentration) and thus plays a key role in regulating 5-HT neurotransmission [27]. The decrease in mRNA* Tph2* level may indicate the involvement of alpha-tocopherol in regulating 5-HT neurotransmission. It is known that the antidepressant-like effect of *α*-tocopherol involves, at least in part, the modulation of serotonergic, dopaminergic, and noradrenergic systems. It was also shown that excess inflammatory cytokines in the nervous system disturb the production of serotonin [50]. Therefore, we suggest that the anti-inflammatory effect of *α*-tocopherol plays a key role in the modulation of* Tph2* gene expression.

Our results also indicated that *α*-tocopherol supplementation prevented the increase of GSSG and increased the GSH/GSSG ratio in rats after exposure to chronic stress. At the same time, there was a decrease in GR activity and TBARS level. And although, as discussed above, our observations do not confirm the occurrence of oxidative stress in the blood of rats after exposure to chronic stress, the results indicate that *α*-tocopherol supplementation seems to have a beneficial effect in this regard. The dose of *α*-tocopherol (100 mg/kg) used in our study was selected based on available data from the literature indicating its antioxidant activity in rats [51–53]. Interestingly, it was noted that supplementation with *α*-tocopherol reduces IL-6 and TNF-*α* levels in the blood of animals after exposure to chronic stress. As mentioned, the induction of these cytokines may generate ROS under proinflammatory conditions and consequently lead to cell damage. Inflammatory stimuli such as excessive ROS produced in the process of oxidative metabolism were reported to initiate the inflammatory process resulting in secretion of proinflammatory cytokines [54]. In this context, a decrease in IL-6 and TNF-*α* concentration may suggest that the cells are protected from excessive harmful effects of ROS through anti-inflammatory effects. Thus, data obtained in our study can suggest the potential benefits of *α*-tocopherol supplementation under chronic stress conditions.

## 5. Conclusions

To summarize, biochemical analysis does not confirm the occurrence of oxidative stress in the blood of rats after exposure to CVS. However, the analysis of the expression of the* Fkbp5* and* Tph2* genes indicates the occurrence of a depressive state in the brain of rats after exposure to chronic variable stress, which does not confirm diagnostic utility of oxidative stress parameters in the blood. A significant increase in plasma IL-6, IL-1*β*, and TNF-*α* concentrations indicates the occurrence of the inflammatory processes after exposure to chronic stress, probably in response to oxidative stress. This thesis is supported by results obtained in the CVS+*α*-tocopherol group, where the supplementation with *α*-tocopherol reduces IL-6 and TNF-*α* levels, which suggests a relationship between redox equilibrium disorders and inflammatory response. This assumption is further enhanced by amelioration in* Fkbp5 *and* Tph2* genes expression in group CVS supplemented with *α*-tocopherol compared to the CVS rats. Subsequent studies are needed to verify beneficial effect of *α*-tocopherol on pharmacological depression therapy with classical antidepressant.

## Figures and Tables

**Table 1:** The stressors used during the chronic variable stress (CVS) procedure.

Day of experiment	Stress factor	Time of duration	Start time
1	water deprivation	24 h	8:00 a.m.
2	food deprivation	24 h	9:00 a.m.
3	isolation	24 h	10:00 a.m.
4	isolation	24 h	10:00 a.m.
5	isolation	24 h	10:00 a.m.
6	flashing light	3 h	12:00 a.m.
7	food deprivation	24 h	8:00 a.m.
8	forced swimming	10 min	9:00 a.m.
9	restraint	1 h	11:00 a.m.
10	water deprivation	24 h	9:00 a.m.
11	no stressor	–	–
12	no stressor	–	–
13	restraint and cold	2 h	10:00 a.m.
14	flashing light	2.5 h	9:00 a.m.
15	food deprivation	24 h	8:00 a.m.
16	forced swimming	15 min	12:00 a.m.
17	isolation	24 h	8:00 a.m.
18	isolation	24 h	8:00 a.m.
19	isolation	24 h	8:00 a.m.
20	water deprivation	24 h	10:00 a.m.
21	food deprivation	24 h	9:00 a.m.
22	flashing light	3 h	13:00 a.m.
23	restraint	2 h	12:00 a.m.
24	isolation	24 h	8:00 a.m.
25	isolation	24 h	8:00 a.m.
26	restraint and cold	1.5 h	12:00 a.m.
27	forced swimming	10 min	10:00 a.m.
28	flashing light	3.5 h	8:00 a.m.
29	no stressor	–	–
30	food deprivation	24 h	8:00 a.m.
31	restraint	3 h	9:00 a.m.
32	flashing light	2 h	10:00 a.m.
33	water deprivation	24 h	8:00 a.m.
34	restraint and cold	2 h	10:00 a.m.
35	forced swimming	15 min	11:00 a.m.
36	isolation	24 h	8:00 a.m.
37	isolation	24 h	8:00 a.m.
38	no stressor	–	–
39	flashing light	3 h	13:00 a.m.
40	forced swimming	10 min	8:00 a.m.

**Table 2:** Gene symbols, gene names, GenBank reference sequence accession numbers, and TaqMan Assays IDs used in this study for qPCR.

Gene symbol	Gene name	GenBank RefSeq Accession Number	Assay ID
*Fkbp5*	FK506 binding protein 5	NM_001012174.1	Rn01768371_m1
*Tph2*	tryptophan hydroxylase 2	NM_173839.2	Rn00598017_m1

NM_001012174.1 Rattus norvegicus FK506 binding protein 5 (*Fkbp5*), mRNA; NM_173839.2 Rattus norvegicus tryptophan hydroxylase 2 (*Tph2*), mRNA.

## Data Availability

The data used to support the findings of this study are available from the corresponding author upon request.
